# Symmetry Breaking as an Interdisciplinary Concept Unifying Cell and Developmental Biology

**DOI:** 10.3390/cells10010086

**Published:** 2021-01-07

**Authors:** Andrew B. Goryachev

**Affiliations:** SynthSys, Centre for Synthetic and Systems Biology, Institute for Cell Biology, University of Edinburgh, Edinburg EH9 3BD, UK; Andrew.Goryachev@ed.ac.uk

The concept of “symmetry breaking” has become a mainstay of modern biology, yet you will not find a definition of this concept specific to biological systems in Wikipedia. The term first appeared in the early 1960s in theoretical particle physics and rapidly spread throughout the entire domain of physics. When explaining symmetry breaking, physicists like to paint a mental image of a ball precariously perched on the very top of a bump separating two identical wells. The slightest noise is sufficient to push the ball off the top and into either of the wells. After that, only one of them has the ball, and thus the initial unstable symmetry of the system is broken. While visually appealing, the allegory of a ball in a well is not always easy to relate to biology. Furthermore, throughout development, biological organisms often seem to transit from an amorphous asymmetric state, e.g., a clump of dividing cells known as a morula, to the state with a striking apparent symmetry, such as mature hydra polyp. This apparent increase in the symmetry of developing organisms can be misinterpreted as contradicting the applicability of the concept of symmetry breaking in biology. 

History can help us resolve this conundrum. The first mention of symmetry breaking in regards to biological and chemical systems dates back, presumably, to the seminal paper by Prigogine and Nicolis [1], who stated that the pattern-forming mechanism proposed by Turing to explain biological morphogenesis [2] is “*symmetry breaking* as it leads from a homogeneous to an inhomogeneous steady state”. In the mind of a theoretical physicist, a spatially homogenous chemical system is akin to an unbounded featureless plane, and any geometric translation, mirror reflection, or rotation of such a plane transforms it back into itself, defining it as infinitely symmetric. The appearance of a pattern (such as a hexagonal lattice of Turing spots) breaks this symmetry, leaving few if any geometric degrees of freedom that can transform the now patterned system back into itself. Similarly, an unpolarized cell can be modeled by a perfect sphere, which remains itself after any rotation around any axis that passes through its center. Cellular polarization effectively selects only one such axis around which a cell can be rotated so it is still “transformed into itself”. Multicellular development is even harder to interpret in geometric terms, although specific examples of such geometric symmetry-breaking transitions can be identified. Thus, similar to cellular polarization, gastrulation reduces the spherical symmetry of the blastula to the cylindrical symmetry of the gastrula. Instead of looking for specific broken symmetries, it might be more productive to follow in the footsteps of Prigogine and Nicolis and consider all phenomena of biological pattern formation as manifestations of symmetry breaking. This pluralistic approach conceptually unifies efforts to understand biological morphogenesis on both subcellular and multicellular levels and continues to gain popularity [3].

In the interdisciplinary spirit established by the founding fathers of the field, this Special Issue “Symmetry Breaking in Cells and Tissues” presents a collection of 17 reviews, opinions, and original research papers contributed by theoreticians, physicists and mathematicians, as well as experimental biologists, united by the common excitement about biological pattern formation and morphogenesis. In this issue, the contributors discuss diverse manifestations of symmetry breaking in biology and showcase recent developments in experimental and theoretical approaches to biological morphogenesis and pattern formation on multiple scales.

Establishment of cell polarity is, perhaps, one of the best-studied manifestations of symmetry breaking in biology. Unicellular model organisms, yeasts, have been particularly useful for studies of cell polarity due to their ease of genetic manipulation and the spectacular cell surface-localized pattern—the conspicuous micron-scale polarity cluster organized by the activity of Cdc42 and other small GTPases from the Ras and Rho families [4,5,6]. Moran and Lew discuss the role of differential diffusion of proteins on the plasma membrane in the establishment of cell polarity in budding yeast [7]. Martin and colleagues deploy optogenetics to study the mechanism of Cdc42 cluster formation in fission yeast and suggest the existence of mechanisms inhibiting de novo formation of the polarity cluster on the cell sides [8]. Khalili, Vavylonis, and colleagues continue the topic of fission yeast cell polarity by elaborating a detailed biophysical model that describes not only static but also spatially oscillating patterns of Cdc42 activity [9]. Finally, Daalman et al. bring up an evolutionary aspect of the budding yeast cell polarity network [10].

Par protein systems constitute another fundamental cell polarity paradigm found in diverse cell types of higher eukaryotes [11,12,13,14]. While much has been learned about the Cdc42 polarity in fungi and Par polarity in *C. elegans* embryos and epithelial cells, understanding the interplay between these two modules has been notoriously difficult. Seirin–Lee, Gaffney, and Dawes construct and analyze a heuristic model of *C. elegans* embryo polarity to explain the interaction between these two modules [15]. A third fundamental paradigm of cell polarity, the polarization of motile chemotactic cells, is revisited by van Haastert, who proposes a unified model of amoeboid movement applicable to both fast- and slow-moving cells [16]. The topic of cell polarity is rounded off by a comprehensive review of published polarity models by Othmer and colleagues [17].

Recent years have seen a dramatic rise of new topics establishing the mechanisms of biological symmetry breaking, which are distinct from the diffusion-driven instability of the reaction–diffusion systems proposed by Turing. Notable examples of these are mechanical instabilities in active systems consisting of cytoskeletal polymers and molecular motors [18,19] and protein phase separation [20,21]. In this Special Issue, Schwille and colleagues present an in vitro reconstitution of actomyosin cortices, in which they observe symmetry breaking and emergence of directed flows [22]. The theme of actomyosin contractility continues in the contribution from Gerisch and colleagues who discuss unilateral ingression of cleavage furrows in multinucleated cells of *Dictyostelium* amoeba [23]. The theme of protein phase separation and its role in the establishment of cellular memory and stress adaptation is reviewed by Caudron and colleagues [24].

Symmetry breaking on the scale of tissues and organs transcends multiple fields of developmental biology and is an area of research that has both a distinguished past and a rapidly developing present [25,26,27,28]. Connecting unicellular and multicellular scales, Yap and colleagues review epithelial cell extrusion, a form of collective behavior of cells within epithelial sheets that plays an important role in normal morphogenesis and development of cancer [29]. A review by Manceau, Bailleul, and Touboul provides an extensive overview of mechanisms and models of multicellular pattern formation [30].

Several contributions focus on theoretical aspects of pattern formation and morphogenesis. Naoz and Gov extend the theme of the pattern-forming role of proteins capable of bending the cell membrane and provide analysis of actin-mediated protrusions on the ventral side of adhered cells [31]. They show that facing hard substratum and adhering to it can stabilize such protrusions or induce their transition into propagating wavelike structures. Formation of protein patterns on the cell membrane in the presence of flows in the cytoplasm or cellular cortex is the focus of contribution by Frey and colleagues, who show that such flows can induce interesting and nontrivial effects modulating protein patterns [32]. An important theoretical question in the field of cellular pattern formation is whether specific mechanisms are required to produce a single structure, such as the one necessary for cellular polarization, or a multitude of similar structures, such as podosomes or microvilli. Goryachev and Leda discuss recent theoretical work exploring this question in the minimal mass-conserved activator–substrate models and conclude that the choice between competition and coexistence of structures is determined by the complex interplay of multiple system parameters, rather than by the type of the molecular mechanism [33]. Developing this theme further, Banerjee and colleagues consider induction of multiple structures in dynamically growing systems [34]. Finally, Beta, Gov, and Yochelis discuss a dynamic pattern, representative of intracellular actin waves, in a minimal activator–inhibitor model with mass conservation [35].
